# Preventing germ cell death by inactivating aryl hydrocarbon receptor (AHR)

**DOI:** 10.1038/cddis.2016.20

**Published:** 2016-02-25

**Authors:** P Esakky, K H Moley

**Affiliations:** 1 Department of Veterans Affairs Medical Center, St. Louis, MO, USA; 2 Department of Obstetrics and Gynecology, Washington University School of Medicine, St. Louis, MO, USA

Tobacco causes nearly six million deaths per year worldwide; in the US, smoking and second-hand smoke exposure causes one in every five deaths and incur almost $300 billion annually in total economic costs.^1^ Approximately 35% of men of reproductive age in the US smoke cigarettes, affecting not only themselves but also the environment and their progeny. Smoking causes infertility in men, and children born to male smokers are at increased risk of childhood cancers and birth defects, such as cardiac defects, cleft palate, renal agenesis, and spina bifida. These effects are due to the accumulation of toxic constituents of cigarette smoke (CS) in the systemic circulation and seminal plasma.^2^ CS contains more than 7000 chemicals, including at least 539 polycyclic aromatic hydrocarbons (PAHs), of which 69 are proven carcinogens that are also products of incomplete combustion of fossil fuels and environmental pollution.^3^

PAHs such as dioxins, and benzopyrene in cigarette smoke condensate (CSC) or tar, exert their cytotoxicity by activating the ligand-inducible transcription factor, aryl hydrocarbon receptor (AHR).^4^ Whereas the endogenous activation of AHR by growth factors and dietary metabolites like kynurenic acid maintains tissue homeostasis and regulation of normal immune function, its exogenous activation by PAHs leads to spermatogenic arrest, suppression of meiosis, cell cycle arrest, spermatocyte cell death, and male infertility.^5, 6^ However, the precise role of AHR in spermatocyte susceptibility to CSC is still emerging. In an earlier study,^7^ we demonstrated that the CSC induced several molecular and phenotypic changes in the spermatocyte cell line, (GC-2spd(ts)).^8^ By using a competitive AHR-specific pharmacological inhibitor, CH223191,^9^ we observed that many of the gene expression changes were dependent on AHR signaling. In the subsequent work, we showed that inhibition of AHR abrogated cell cycle arrest in spermatocytes.^10^ However, given that AHR is required for normal sperm development in mice,^11^ our recent report published in *Cell Death Discovery*^12^ compared the effects of pharmacological inhibition of AHR to knockdown of AHR expression, yielding intriguing findings (Figure 1).

We first characterized the effects of CSC on GC-2spd(ts) spermatocytes and found that CSC caused oxidative stress both in the cytoplasm and the mitochondria, though it did not affect mitochondrial membrane potential. Additionally, CSC treatment of spermatocytes promoted apoptosis, as indicated by increased expression of both anti- and pro-apoptotic genes, DNA damage, activation of caspases 3 and 7, and plasma membrane damage. Others have reported similar results in different cell types.^13^ Pretreatment of the cells with CH223191 significantly reduced several of these effects, including DNA damage, caspase activation, and membrane alteration.

To compare the effects of AHR inhibition to reduction of AHR expression, we used siRNA to knockdown expression of *Ahr* in spermatocytes by 70%. Strikingly, we observed that *Ahr* knockdown did not abrogate many of the CSC-mediated effects that were suppressed by AHR inhibition. For example, *Ahr* knockdown did not reduce CSC-mediated caspase activation or plasma membrane damage. Moreover, spermatocytes in which *Ahr* was knocked down had higher levels of DNA damage than control spermatocytes. This finding suggests a regulatory role for AHR in the early stages of DNA damage or repair^7^ and supports an earlier finding that loss of AHR triggers DNA damage.^14^ To confirm these findings, we compared control and *Ahr*-null mouse embryonic fibroblasts (MEFs) and found that complete loss of *Ahr* was unable to prevent the CSC-mediated increase in caspase activation and membrane damage.

We demonstrated earlier^10^ that the cross talk between AHR–NRF2 pathway and MAPK signaling is required for CSC-induced spermatocyte cell death. However, here we found that MAPK inhibitors could not prevent CSC-induced membrane damage in spermatocytes. We interpret this finding to mean that the membrane damage is directly caused by the CSC-induced increase in free oxygen radicals and the involvement of other signaling mediators in addition to the MAPK pathway as reported by others in rat lung.^15^ Our results reveal that pharmacological inhibition and genetic manipulation of AHR have different effects; this fact should be kept in mind in future studies. More importantly, our data indicate that inhibition of AHR by using drugs such as CH223191 may be a viable prophylactic strategy to prevent germ cell death mediated by exposure to CS and other environmental pollutants containing PAHs.

## Figures and Tables

**Figure 1:**
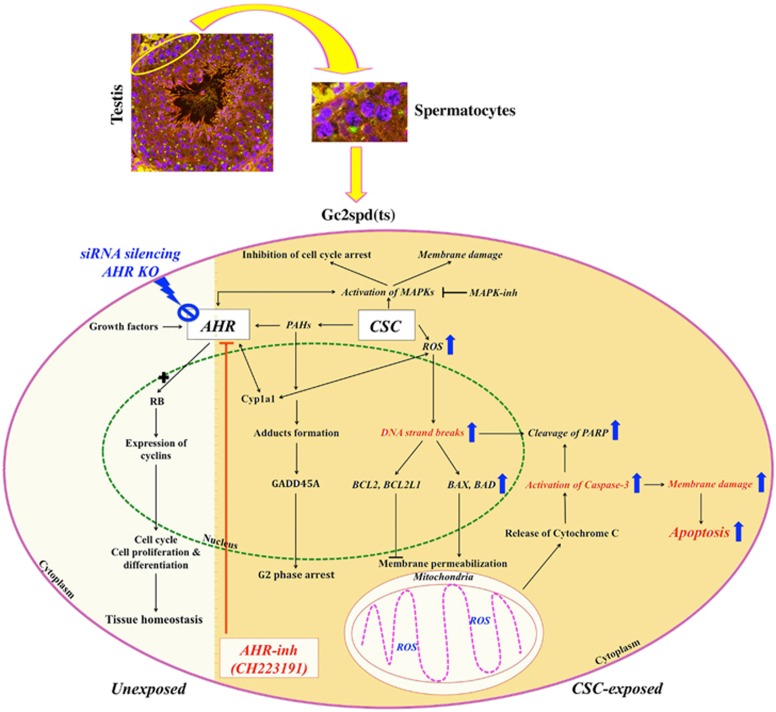
A schematic representation of proposed CSC-mediated cell death regulation through AHR in spermatocytes. Growth factors maintain tissue homeostasis through AHR, which promotes expression of cyclins by forming growth-stimulating complex with molecules such as retinoblastoma (unexposed). On the other, CSC via PAHs mediates cross talk between AHR and MAPKs. Inhibition of activated MAPKs abrogates cell cycle arrest but fails preventing membrane damage. Pretreatment with AHR-inh (CH223191) attenuates CSC-induced DNA strand breaks, caspase activation, and membrane alteration (events marked in red). However, genetic silencing of *Ahr* either by siRNA knockdown in spermatocytes or absence of *Ahr* in knockout MEF elevates ROS, enhances expression of pro- and anti-apoptotic proteins, and promotes apoptotic cascade upon exposure to CSC (marked with blue arrow). Genetic manipulation and pharmacological inhibition distinguishes the protective role of AHR in maintaining normal cellular homeostasis and advocates AHR as a potential prophylactic therapeutic target to promote cell survival and growth under CS-exposed environment, respectively. CSC-exposed is colored yellow on right and findings of this study are italicized. MAPKs, mitogen-activated protein kinases; ROS, reactive oxygen species

## References

[bib1] USDHHS.The Health Consequences of Smoking—50 Years of Progress: A Report of the Surgeon General. USDHHS: Atlanta, GA, USA, 2014.

[bib2] Mostafa M. J Adv Res 2010; 1: 179–186.

[bib3] Rodgman A, Perfetti TA. The Chemical Components of Tobacco and Tobacco Smoke. CRC Press, Taylor & Francis Group: Boca Raton, FL, USA, 2009.

[bib4] Gu YZ et al. Annu Rev Pharmacol Toxicol 2000; 40: 519–561. 10836146 10.1146/annurev.pharmtox.40.1.519

[bib5] Georgellis A et al. Mutation Res 1990; 231: 125–135. 2117249 10.1016/0027-5107(90)90019-z

[bib6] Gaspari L et al. Mutation Res 2003; 535: 155–160. 12581533 10.1016/s1383-5718(02)00297-8

[bib7] Esakky P et al. Reprod Toxicol 2012; 34: 665–676. 23069111 10.1016/j.reprotox.2012.10.005

[bib8] Wolkowicz MJ et al. Biol Reprod 1996; 55: 923–932. 8879510 10.1095/biolreprod55.4.923

[bib9] Kim SH et al. Mol Pharmacol 2006; 69: 1871–1878. 16540597 10.1124/mol.105.021832

[bib10] Esakky P et al. J Mol Cell Biol 2015; 7: 73–87. 25548370 10.1093/jmcb/mju049

[bib11] Hansen DA et al. Biol Reprod 2014; 90: 1–12.

[bib12] Esakky P et al. Cell Death Discov 2015; 1: 15050. 10.1038/cddiscovery.2015.50 PMC4979462 27551479

[bib13] Rico de Souza A et al. J Biol Chem 2011; 286: 43214–43228. 21984831 10.1074/jbc.M111.258764 PMC3234839

[bib14] Marlowe JL et al. Mol Biol Cell 2008; 19: 3263–3271. 18524851 10.1091/mbc.E08-04-0359 PMC2488290

[bib15] Kuo W et al. Chemico-Biol Interact 2005; 155: 31–42. 10.1016/j.cbi.2005.04.008 15970277

